# Two Versus Three Narrow-Diameter Implants with Locator Attachments Supporting Mandibular Overdentures: A Two-Year Prospective Study

**DOI:** 10.1155/2012/285684

**Published:** 2012-06-18

**Authors:** Ali M. El-Sheikh, Omar F. Shihabuddin, Sahar M. F. Ghoraba

**Affiliations:** ^1^Department of Prosthetic Dentistry, Faculty of Dentistry, Tanta University, Tanta 31111, Egypt; ^2^Department of Oral Maxillofacial Surgery, Dammam Dental Centre, Dammam Medical Complex, Dammam 31433, Saudi Arabia; ^3^Department of Oral Medicine, Periodontology, Radiology and Diagnosis, Faculty of Dentistry, Tanta University, Tanta 31111, Egypt

## Abstract

*Purpose*. To compare treatment outcome (survival rate, condition of hard and soft peri-implant tissues) and prosthodontic maintenance requirements of two versus three narrow-diameter bone level implants with Locator attachments supporting mandibular overdentures. *Materials and Methods*. Twenty completely edentulous patients with atrophic mandibles were treated. Ten patients (Group A) were treated with overdentures supported by two narrow (3.3-mm diameter) implants (Straumann AG, Basel, Switzerland) and ten patients (Group B) were treated with overdentures supported by three narrow implants. Locator (Zest Anchors, USA) attachments were used for prosthetic anchorage. Standardized clinical and radiographic parameters (survival rate, plaque index, calculus index, gingival index, bleeding index, probing depth and marginal bone loss) were evaluated at the time of the completion of the prosthetic treatment (baseline) and after 6, 12 and 24 months of functional loading. Prosthodontic maintenance requirements were also scored. *Results*. Only one implant was lost (Group B) during the healing period. There were no significant differences with regards to any of the studied clinical and radiographic parameters between the two groups (*P* > 0.05). Few prosthetic complications were recorded. *Conclusions*. No need to insert more than two narrow-diameter bone level implants with Locator attachments in cases of atrophic mandible to support an overdenture, however, long-term prospective studies are required to support this notion.

## 1. Introduction

Edentulism is considered a poor health outcome and may compromise quality of life. Implant-supported overdentures provide a good opportunity for dentists to improve the quality of life and oral health [1]. Atrophic mandible poses a significant challenge to successful oral rehabilitation with dental implants [2]. Although ridge augmentation can help to restore ridge volume, grafting procedures can significantly increase patient morbidity, costs, and treatment time [3–5].

Narrow-diameter implants are generally used for alveolar ridges that are thin for regular implants with a diameter of approximately 4.0 mm to avoid advanced surgical procedures, such as local bone augmentation [6–9]. They are also indicated when the bone deficiency is circumferential around an implant or the interdental space is limited, as in the replacement of mandibular incisors and maxillary lateral incisors [6, 10]. Caution in the use of narrow-diameter implants has been advocated because of the concern regarding the negative impact of loading in these implants, with lower stability when compared to regular platform implants [11], and increased probability of fracture in clinical practice [8]. Moreover, a nonlinear finite element analysis has shown that the neck of the implant represents a potential zone of fracture when subjected to high bending force [12], making it mandatory to increase the implant support to improve the biomechanical outcome of the treatment with narrow-diameter implants [13].

High implant success rates have been achieved by Ahn et al. [14] (96.3%), Griffitts et al. [15] (97.4%), Cho et al. [16] (94%), Morneburg and Pröschel [17] (95.5%), Jofre et al. [18] (100%), Elsyad et al. [19] (96.4%), and Al-Nawas et al. [20] (98%) using narrow-diameter implants to support mandibular overdentures.

The Locator attachment was introduced in 2001. This attachment is self-aligning, has dual retention, and is available in different colors with different retention values [21, 22]. Locator attachments are available in different vertical heights. They are resilient, retentive, and durable and have some built-in angulation compensation. In addition, repair and replacement are easy and fast [23–25].

There is limited evidence for the use of narrow-diameter implants for rehabilitation of the completely edentulous atrophic mandibles with Locator attachments to support overdentures. Therefore, the purpose of this 2-year study was to compare treatment outcome (survival rate and condition of hard and soft peri-implant tissues) and prosthodontic maintenance requirements of two versus three narrow-diameter bone level implants with Locator attachments supporting mandibular overdentures.

## 2. Materials and Methods

### 2.1. Patient Selection

Twenty completely edentulous patients, 11 men and 9 women, ranging from 54 to 68 years of age (mean age 60.4 years) were included in the study. These patients were treated in Dammam Dental Centre, Dammam Medical Complex (Dammam, Saudi Arabia) in the period from March to October 2009. All patients signed an informed consent form. Ethical approval for the project was granted by the Human Research Ethics Committee of the Dammam Medical Complex, Dammam, Saudi Arabia.

Inclusion criteria dictated that the patient is completely edentulous for at least 1 year, has no previous denture experience, and has sufficient bone for an implant of at least 10 mm length and 3.3 mm diameter. Exclusion criteria included any medical condition contraindicating implant surgery, logistic or physical reasons that could affect follow-up, psychiatric problems and disorders to the implant site related to a history of radiation therapy to the head and neck, or bone augmentation.

The patients were informed about the treatment options (overdenture on two or three implants). Treatment was randomly allocated by lots resulting in ten patients (Group A) to be treated with two narrow (3.3 mm diameter) bone level implants (Straumann AG, Basel, Switzerland) and ten patients (Group B) to be treated with three narrow (3.3 mm diameter) Straumann bone level implants.  Table 1 summarizes the characteristics of the patients of the two groups.

### 2.2. Surgical Procedures

Thorough preoperative clinical assessment was carried out for the quantity and morphology of the bone that would host the implants. Preoperative panoramic and periapical radiographs were used for radiographic evaluation of the placement sites to avoid potential complications with important anatomy in these regions.

The components used were narrow bone level implants with a diameter of 3.3 mm and ranged between 10 and 14 mm in length. The distribution of the lengths of the implants is presented in Table 2. Locator attachments (Zest Anchors LLC, Escondido, CA, USA) were used for prosthetic anchorage. In Group A, the implants were placed in the canine region of the mandible, at equal distance from the midline, while, in Group B, one central implant was placed in the midline and the two lateral implants were placed at equal distance from the central implant in the canine regions.

**Figure 1:**
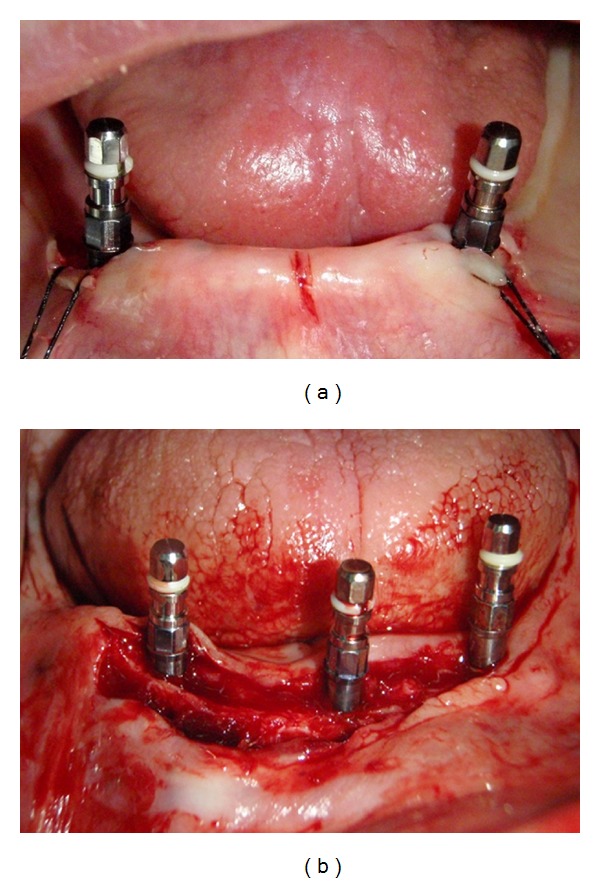
Surgical placement of the implants in the mandible.

One-stage surgical approach was followed throughout the whole study (Figures 1(a) and 1(b)). Under local anaesthesia, a minimal crestal incision (envelope type) was made and a mucoperiosteal flap was raised, both on the labial and the lingual aspects, to enable adequate visualization of the lingual aspect of the mandible and to evenly divide the available keratinized tissue. The osteotomy was prepared using a standard bone drilling protocol, according to the manufacturer's directions. Bone quality was identified, and bone tap was used in types 1 and 2. Initial implant stability was tested manually by hand, and insertion torques ≥35 Ncm were acceptable. Healing abutments of appropriate length were connected, and the mucosa was adjusted and sutured (4-0 Vicryl, Ethicon, Johnson & Johnson, Brussels, Belgium).

Antibiotic (Augmentin 625 mg) and nonsteroidal anti-inflammatory (Ibuprofen 400 mg) medications were given to the patients every 8 hours for 5 days postoperatively. All patients were limited to a soft diet for 10 days. The patients were instructed in a plaque control protocol at the time of implant placement, and this was reinforced at subsequent reviews.

### 2.3. Prosthetic Procedures

**Figure 2:**
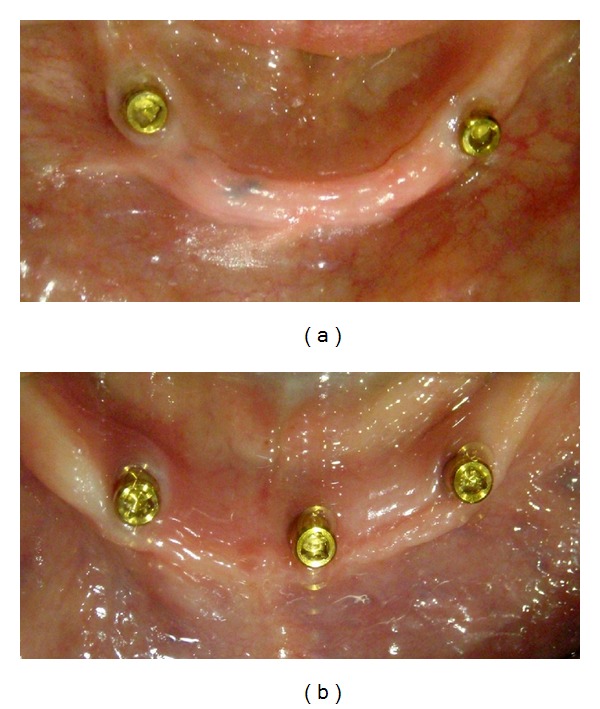
Locator attachments screwed into the implants 10 weeks after their placement.

**Figure 3:**
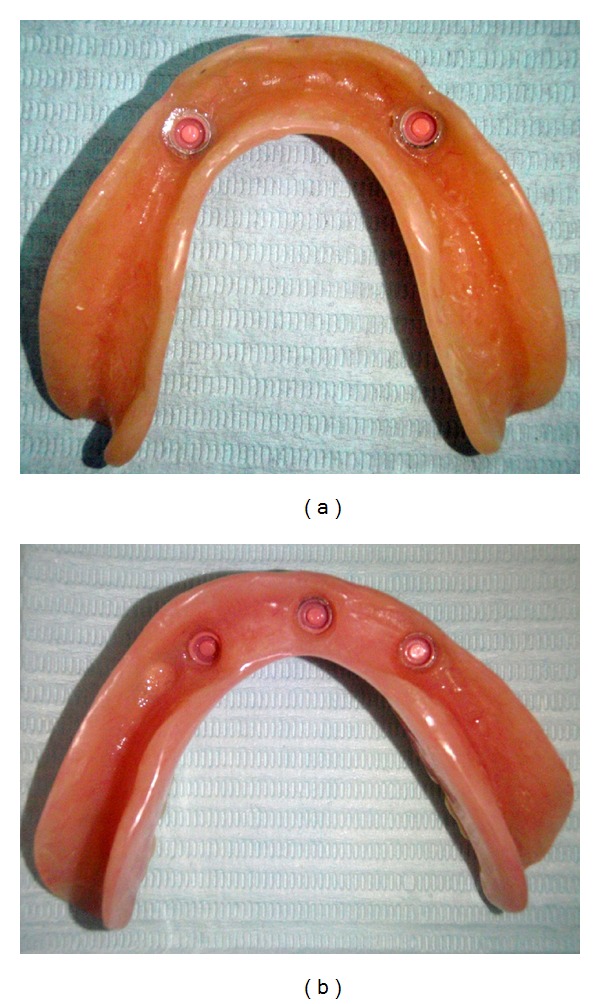
Finished mandibular overdentures with pink replacement males immediately before insertion.

The healing abutments were replaced by Locator attachments (Figures 2(a) and 2(b)) 10 weeks after implant placement. A torque of 35 Ncm was used for tightening the attachments. The height of the attachments (ranging from 2 to 6 mm) was selected according to the height of the gingiva. The selection was carried out with the aid of the periodontal prob. Preliminary impressions for maxillary and mandibular arches were taken with stock trays using irreversible hydrocolloid (Hydrogum, Zhermack, Italy). Secondary impressions were taken with autopolymerized acrylic resin special trays using vinyl polysiloxane impression material (Express, 3 M ESPE Dental Products, USA). Record blocks were fabricated on the duplicates of the master models for jaw registration. Teeth try-in and manufacturing of the acrylic dentures were carried out using standard prosthetic procedures. The final prostheses were checked in the patient's mouth and the required adjustments were carried out. The denture caps with attached black processing males were connected to the mandibular denture using the indirect technique on the mandibular master model. The black processing males were removed after polishing the denture, and the appropriate Locator replacement males were inserted according to the retention required. The maxillary complete denture and implant-retained mandibular overdenture (Figures 3(a) and 3(b)) were delivered to the participants approximately 12 weeks after implant placement. 

### 2.4. Clinical Analysis

The clinical analysis included a number of parameters. Loss of implants was scored after removal of a loose implant any time after placement. For the presence of plaque, the index according to Mombelli et al. [26] was used (score 0: no detection of plaque; score 1: plaque can be detected by running a probe across the smooth marginal surface of the attachment and implant; score 2: plaque can be seen by the naked eye; score 3: abundance amount of plaque). The presence of calculus (score 1) or the absence of calculus (score 0) was recorded. To assess potential peri-implant inflammation, the gingival index was used according to the modified Löe and Silness index [27] (score 0: normal peri-implant mucosa; score 1: mild inflammation, slight change in color, and slight edema; score 2: moderate inflammation, redness, edema, and glazing; score 3: severe inflammation, marked redness and edema, and ulceration). For bleeding, the bleeding index according to Mombelli et al. [26] was used (score 0: no bleeding when using a periodontal probe; score 1: isolated bleeding spots visible; score 2: a confluent red line of blood along the mucosal margin; score 3: heavy or profuse bleeding). Probing depth was measured at four sites of each implant (mesially, labially, distally, and lingually) by using a periodontal probe. The distance between the marginal border of the mucosa and the tip of the periodontal probe was scored as the probing depth.

### 2.5. Radiographic Analysis

Standardized intraoral radiographs using a long cone technique of each implant were obtained as described by El-Sheikh et al. [28] To provide a geometrically reproducible alignment, an index was recorded for each patient on the inserted mandibular overdenture with the use of vinyl siloxane material. With the aid of Hawe's sensor holder system (Kerr, KerrHawe SA, Switzerland), the radiographs were taken using direct digital imaging system (Trophy RVG, William Green Pty Ltd, Australia). Images were displayed on a computer screen with such a dimension and brightness that the observer could read comfortably and accurately the image. On each image, the implant-Locator interface and the first bone-to-implant contact were identified and marked with a cursor on the mesial and distal sides of the implant. The analysis program calculated and reported the distance between the two points with a degree of accuracy of ±0.01 mm. The same procedure was performed with all of the follow-up radiographs. The initial postoperative radiographs immediately after insertion of the final overdentures (baseline radiography) were compared with the follow-up radiographs after 6, 12, and 24 months of functional loading. The vertical bone loss was calculated by subtracting the bone heights in the baseline radiographs from those of follow-up radiographs. Data were collected blindly by one experienced observer throughout the entire study.

### 2.6. Postinsertion Maintenance

Any prosthodontic complications/interventions during the 2-year follow-up were recorded according to following events: Locator attachment loosening, retention loss, overdenture repair, overdenture relined/rebased, and opposite denture remade/rebased. 

### 2.7. Data Collection

The data collection (clinical and radiographic outcomes, and prosthodontic maintenance requirements) of all patients was performed as follows: at the completion of the prosthetic treatment (baseline) and after 6, 12, and 24 months of functional loading.

### 2.8. Statistical Analysis

Probing depth was measured at four sites around each implant and bone height measurement was taken mesially and distally on the radiograph for each implant and the mean was taken.

The data were analyzed using *t*-tests for the continuous data and Mann-Whitney tests for the ordinal data. The correlation was tested using Pearson's correlation tests (SPSS for Windows, version 10.0, SPSS Inc., Chicago, IL, USA). In all tests, a significance level of 0.05 was chosen.

## 3. Results

### 3.1. Clinical Parameters

During the healing period prior to the Locator connection operation, one implant (10 mm long) was lost in group B. After removal of the implant and a bone healing period of 6 months, another implant was successfully placed; this patient was included in the study for follow-up evaluation. During the functional period, none of the implants were lost. Survival rate of the narrow-diameter Straumann bone level implants after 2 years is 98%.

The mean scores for the indices of plaque, calculus, gingival, and bleeding were low at all evaluation periods (Table 3). No significant differences between the two- or three-implant concepts were observed with regard to plaque, calculus, gingival, and bleeding scores throughout the observation period (Mann-Whitney test *P* > 0.05). No significant difference between both groups was observed with regard to probing depth (*t*-test, *P* > 0.05) (Table 3).

### 3.2. Radiographic Parameter

The marginal bone loss as a function of time is shown in Table 4. All implants showed less than 1 mm of marginal bone loss during the first-year of the follow-up period. The average bone loss over the first year was 0.5 and 0.6 mm for groups A and B, respectively. The average bone loss at the end of the two years follow-up was 0.8 and 0.8 mm for groups A and B, respectively. Therefore, the average bone loss over the second year of follow-up was only 0.3 and 0.2 mm for groups A and B, respectively. No significant differences in bone loss were observed between both groups (*t*-test, *P* > 0.05). There was no significant difference with regard to peri-implant bone loss between lateral and central implants in group B during the evaluation period. There was no correlation between the radiographic findings and the peri-implant clinical parameters (Pearson's correlation test, *P* > 0.05).

### 3.3. Prosthetic Maintenance

Very few prosthetic complications were recorded in both groups. No loosening of the Locator attachments was recorded throughout the follow-up period. The retention values were increased after 12 months in only two cases of group A by replacing the pink replacement males with the clear ones. No repair of the overdentures bases was required in both groups. There was no need for relining the overdentures in group B, while only one overdenture required relining in group A after 18 months of follow-up. Maxillary denture rebasing was required in only one patient in group A and in two patients in group B.

## 4. Discussion

In cases where bone width is narrow, local bone augmentation to enable the use of standard-size implants is an option. Augmentation techniques increase the treatment time and costs and are invasive. The main advantage of the narrow-diameter implants is the ability to apply less invasive surgical procedures when there is circumferential bone deficiency around the implants. A reduced diameter means a reduction in the contact surface between the implant and the bone, and one might ask whether osseointegration is sufficient to withstand loading forces. Decreasing the diameter also means increasing the risk of implant fracture due to reduced mechanical stability and increasing the risk of overload.

It has been suggested that narrow-diameter implants are less prone to stand against stress structurally and could increase the stress transmitted to the bone [29–31]. For example, it was estimated that fracture resistance of the implant decreases approximately 25% when implant diameter reduces from 3.75 to 3.3 mm [29]. In the present study, no implant fractures were recorded during the follow-up period.

The 2-year survival rate of narrow-diameter Straumann bone level implants in this study is 98%. This percentage is comparable with other clinical studies, which have reported survival rates of narrow-diameter implants supporting mandibular overdentures ranging from 94% to 100% [14–20]. These findings support the hypothesis that narrow-diameter implants can be used in prosthetic rehabilitation of the atrophic mandibles with predictable positive outcomes.

The mean indices for plaque, calculus, gingival, and bleeding were low at all evaluation periods with no significant difference between the two groups. The strict oral hygiene regime to which the patients were subjected provided healthy peri-implant tissues. These findings are in agreement with other studies [20, 28, 32].

The overall mean marginal bone loss after 1 year of function in the present study was less than 1 mm for both groups which is in agreement with previous studies [19, 20, 33]. The values of marginal bone resorption recorded in the present study over the second year of follow-up were within the accepted standard success criteria for implants [34].

Very few prosthetic complications were recorded during this 2-year study. The findings in the present study are in agreement with the study of Cakarer et al. [1], which reported no prosthetic complications with Locator attachments in comparison with the ball and bar attachments. Alsabeeha et al. [35] reported that Locator attachments of titanium nitride-coated patrices and nylon matrices showed extensive deformation and deterioration with a substantial need for maintenance. Evtimovska et al. [22] demonstrated that retentive values of the Locator attachments are reduced significantly after multiple pull. The retention values were increased after 12 months in two cases of group A by changing the replacement males. There was no need for relining any of the overdentures in group B, while only one overdenture required relining in group A. This could be explained by the fact that the support in group A is mainly soft tissue support. More alveolar bone resorption might have occurred with soft tissue support.

Since there were no significant differences with regard to any of the studied clinical or radiographic parameters of the peri-implant tissues between the two groups, placement of two narrow-diameter bone level implants in the interforaminal region of the atrophic mandible to support an overdenture with Locator attachments seems to be sufficient. The use of three implants should be restricted to patients with dentate maxilla who will have increased bite forces. 

## 5. Conclusions

With the limited observation period and the number of patients included in this study, it may be concluded that the use of narrow-diameter bone level implants appears to be predictable if clinical guidelines are followed and appropriate prosthetic restorations are provided. It may also be concluded that there seems to be no need to insert more than two narrow-diameter implants with Locator attachments in cases of atrophic mandible to support an overdenture; however, further investigations and long-term prospective studies are certainly required to confirm the encouraging results of this clinical study.

## Figures and Tables

**Table 1:** Characteristics of the groups of the study.

Characteristics	Group A (*n* = 10)	Group B (*n* = 10)
Mean age in years (SD)	61.4 (6.3)	58.9 (5.9)

Gender (M/F)	5/5	6/4

Mean edentulous period in years (SD)	1.6 (1.2)	2.1 (1.1)

**Table 2:** Distribution of lengths of inserted implants.

	Length (mm)			Total
	10 mm	12 mm	14 mm	
Group A (*n* = 10)	5	12	3	20

Group B (*n* = 10)	6	21	3	30

**Table 3:** Peri-implant parameters (Means and SDs) at all recall examinations.

	Baseline			6 Months			12 Months			24 Months		
	Group A (*n* = 10)	Group B (*n* = 10)	*P*-value	Group A (*n* = 10)	Group B (*n* = 10)	*P*-value	Group A (*n* = 10)	Group B (*n* = 10)	*P*-value	Group A (*n* = 10)	Group B (*n* = 10)	*P*-value
Plaque-index mean (SD)	0.1 (0.4)	0.2 (0.5)	0.243	0.4 (0.5)	0.5 (0.5)	0.639	0.6 (0.6)	0.6 (0.7)	0.843	0.8 (0.9)	0.9 (0.7)	0.482

Calculus-index mean (SD)	0.2 (0.3)	0.3 (0.3)	0.367	0.3 (0.4)	0.3 (0.5)	0.843	0.4 (0.6)	0.4 (0.6)	0.932	0.5 (0.6)	0.4 (0.6)	0.231

Gingival-index mean (SD)	0.2 (0.4)	0.3 (0.5)	0.546	0.4 (0.3)	0.4 (0.6)	0.519	0.5 (0.6)	0.6 (0.7)	0.932	0.6 (0.6)	0.5 (0.4)	0.439

Bleeding-index mean (SD)	0.4 (0.4)	0.5 (0.7)	0.765	0.5 (0.4)	0.7 (0.9)	0.629	0.8 (1.1)	0.9 (0.8)	0.629	1.0 (0.9)	1.1 (0.9)	0.658

Probing-depth mean (SD)	2.9 (0.9)	3.0 (1.1)	0.673	3.2 (0.9)	3.3 (1.1)	0.614	3.4 (1.2)	3.3 (1.0)	0.846	3.2 (0.8)	3.4 (0.7)	0.749

**Table 4:** Results from radiographic measurements.

	Group A (*n* = 10)	Group B (*n* = 10)	*P*-value
Mean loss of marginal bone between baseline and 6 months in mm (SD)	0.3 (0.3)	0.4 (0.3)	0.266

Mean loss of marginal bone between baseline and 12 months in mm (SD)	0.5 (0.2)	0.6 (0.8)	0.214

Mean loss of marginal bone between baseline and 24 months in mm (SD)	0.8 (0.5)	0.8 (0.9)	0.342
